# The effect of data cleaning on record linkage quality

**DOI:** 10.1186/1472-6947-13-64

**Published:** 2013-06-05

**Authors:** Sean M Randall, Anna M Ferrante, James H Boyd, James B Semmens

**Affiliations:** 1 Centre for Data Linkage, Curtin Health Innovation Research Institute, Curtin University, Perth, WA GPO U1987, Australia

**Keywords:** Data cleaning, Data quality, Medical record linkage

## Abstract

**Background:**

Within the field of record linkage, numerous data cleaning and standardisation techniques are employed to ensure the highest quality of links. While these facilities are common in record linkage software packages and are regularly deployed across record linkage units, little work has been published demonstrating the impact of data cleaning on linkage quality.

**Methods:**

A range of cleaning techniques was applied to both a synthetically generated dataset and a large administrative dataset previously linked to a high standard. The effect of these changes on linkage quality was investigated using pairwise F-measure to determine quality.

**Results:**

Data cleaning made little difference to the overall linkage quality, with heavy cleaning leading to a decrease in quality. Further examination showed that decreases in linkage quality were due to cleaning techniques typically reducing the variability – although correct records were now more likely to match, incorrect records were also more likely to match, and these incorrect matches outweighed the correct matches, reducing quality overall.

**Conclusions:**

Data cleaning techniques have minimal effect on linkage quality. Care should be taken during the data cleaning process.

## Background

### Record linkage in context

Record linkage is the process of bringing together data relating to the same individual from within or between datasets. This process is non-trivial when unique person based identifiers do not exist, and linkage is instead performed using probabilistic or other techniques that compare personally identifying information such as name and address, which may include error or change over time.

While record linkage is frequently performed in a business or administrative context to remove duplicate entries from person based datasets, it has also been widely used to enable health researchers to gain event based longitudinal information for entire populations. In Australia, research carried out using linked health data has led to numerous health policy changes [1,2], and the success of previous linkage efforts has led to the development of national linkage infrastructure [3].

### Record linkage methodology

Approaches used in record linkage fall across a spectrum between deterministic and probabilistic methods. Deterministic linkage methods range from simple joins of datasets by a consistent entity identifier to sophisticated stepwise algorithmic linkage which includes additional information to allow variation between records that match i.e. it does not rely on an exact match of the entity identifier. Probabilistic methods, on the other hand, use various fields between data sets to calculate the odds that two records belong together [4]. These odds are represented as probability weights or scores which are calculated (summed) for each pair of records as they are compared. If the total score for a record pair is greater than a set matching threshold, then they are deemed to be a match – the records belong to the same person. The probabilistic approach allows for inconsistencies between records with missing matches i.e. it has the capacity to link records with errors in the linking fields.

Several studies have demonstrated that probabilistic linkage techniques are more robust against errors, and result in better linkage quality than deterministic methods [5-7]. Probabilistic methods are also more adaptable when large amounts of data require linkage [8].

### Data cleaning in record linkage

Irrespective of which linkage approach is being used, the linkage process is usually preceded by a data cleaning phase. Data cleaning (sometimes called standardisation or data cleansing) involves correcting, removing or in some way changing fields based on their values. These new values are assumed to improve data quality and thus be more useful in the linkage process.

There is evidence that improvements in the quality of the underlying data lead to improvements in the quality of the linkage process. For example, early studies of probabilistic linkage in health research demonstrated that greater amounts of personal identifying data greatly improved the accuracy of linkage results [9,10]. Studies have also shown that data items with more discriminating power lead to better linkage results [11,12].

In the absence of strongly identifying personal information, data cleaning has been recognised as one of the key ways to improve the quality of linkage [13]. The record linkage literature identifies data cleaning as one of the key steps in the linkage process [14-17], which can take up to 75% of the effort of record linkage itself [18].

### Data cleaning techniques

A variety of data cleaning techniques are used in record linkage [18-20]. Some data cleaning techniques seek to increase the number of variables by splitting apart free text fields. Others seek to simply transform variables into a specific representation, without actually changing the information. Further techniques aim to change the information in the fields, either by removing invalid values, changing values, or imputing blank values. Based on a review of five institutions conducting linkage in Australia and eight linkage software packages [19], the following data cleaning techniques were identified.

### Reformatting values

Data values can be simply changed to a new format without actually creating or removing information. This ensures that all data is in a common standard for comparison during linkage. For example, two datasets which store dates in a different format (such as ‘11/08/86’ and ‘11^th^ August 1986’), would need to be changed to a common format for comparison. No data is changed by this transformation, only the representation of the data. This technique is essential for ensuring matching fields can be compared [18].

### Removing punctuation

Unusual characters and punctuation are typically removed from alphabetic variables. Names with spaces, hyphens or apostrophes may be more likely to be misrepresented, and removing these values can remove any differences between these values.

### Removing alternative missing values and uninformative values

Datasets can often contain specially coded input values when no information is available – for instance ‘9999’ for a missing postcode. Other datasets may contain information that is not useful to the linkage process - hospital admission records may contain ‘Baby of Rachael’ in a forename field, or ‘NO FIXED ADDRESS’ in an address field. These are commonly removed [18]. In traditional probabilistic linkage, two variables that agree on a value (for instance, both are marked ‘UNKNOWN ADDRESS’) will receive a positive score, which in this case, may be inappropriate. A comparison involving a missing or blank value will typically not result in any positive or negative score.

### Phonetic encoding

By creating an encoding of the phonetic information encapsulated in an alphabetic variable (such as a surname) names that are recorded as different spellings but sound the same will be brought together. Phonetic encoding is a common technique in record linkage. Common encoding algorithms used in record linkage include Soundex [21], NYSIIS [22] and Metaphone [23]. NYSIIS has been used for record linkage in Canada [13], while in the Oxford Record Linkage Study the Soundex value of the NYSIIS code is used in their linkage [18].

### Name and address standardisation

Name standardisation or name parsing is the process of breaking down a person’s full name into its individual components. For instance, a name field with the entry ‘Dr John Harry Williams’ could be broken down into title, first name, middle name and last name, and these components could be individually compared.

Similarly, an address can be broken down into its constituents such as street number, street name and street type. By creating multiple variables in this way, small differences between records such as a different order may have less effect in bringing these records together. Typically the process of breaking the address into separate components has been carried out using a set of rules [24], but the application of statistical methods has also proved useful [25].

### Nickname lookups

A nickname file, containing common nicknames and diminutive names for given names can be used to translate forenames to a common value. Using a nickname lookup, a person recorded as Bill on one dataset and William on another could be given the same first name, potentially bringing these records together [18].

### Sex imputation

A record with a missing sex value can have this value imputed based on their first name. This requires a lookup table which equates common first names with sex.

### Variable and field consistency

Records containing variables which are inconsistent can be edited to remove this inconsistency [20]. For instance, a record with suburb of Sydney and postcode of 6000 is inconsistent, as this is the incorrect postcode for this suburb. It is not often clear which variable to change in order to resolve this inconsistency.

### Prevalence of data cleaning

These techniques encapsulate those found in linkage software packages or in use by dedicated linkage units in Australia during our environmental scan. All techniques listed here were either in use or under consideration by at least one institution conducting linkage in Australia, and all institutions asked used at least one of these techniques to clean their data.

A review of the data cleaning features found in linkage software packages can be found in Table 1. These linkage packages vary from enterprise level commercial packages (IBM’s QualityStage [26]), smaller commercial packages (Linkage Wiz [27] and the now freely available Choicemaker [28]), free university developed software (Febrl [29], FRIL [30], The Link King [31]) and government developed software obtained for evaluation (LINKS [32], BigMatch [33]). Linkage engines are probabilistic (BigMatch, FRIL, Linkage Wiz, FEBRL) a combination of both rules based and probabilistic (LINKS, Link King) or using modern machine learning techniques (ChoiceMaker, FEBRL). Nearly all packages implement data cleaning as a set of functionality which the operator can choose to apply on specified variables in a dataset. In some packages (for instance, The Link King) data cleaning is performed as an automated part of linkage itself, with the operator having little manual control over the steps taken.

**Table 1:** Availability of data cleaning functionality across a sample of linkage packages

	**Linkage Wiz**	**Febrl**	**BigMatch**	**Link king**	**FRIL**	**LINKS**	**ChoiceMaker**	**QualityStage**
Reformat values	Yes	Yes	No	Yes	Yes	No	Yes	Yes
Remove punctuation	Yes	Yes	No	Yes	Yes	No	Yes	Yes
Remove alt. missing values	Yes	Yes	No	Yes	Yes	No	Yes	Yes
Phonetic encoding	Yes	Yes	No	Yes	Yes	No	Yes	Yes
Name/Address Standardisation	Yes	Yes	No	No	No	No	Yes	Yes
Nickname lookup	Yes	Yes	No	Yes	No	No	No	Yes
Sex imputation	Yes	Yes	No	Yes	No	No	No	Yes

Data cleaning functionality in linkage software packages ranges from non-existent (BigMatch, LINKS) to comprehensive (Febrl. QualityStage, Linkage Wiz). Techniques available for reformatting variables typically include trimming, splitting and merging fields, classifying values, and reformatting dates.

Packages which remove specific values typically use a default invalid value list, which can then be added to by the user (for example Febrl, Link King, QualityStage, Linkage Wiz). Phonetic encoding algorithms available typically include Soundex at a minimum, with NYSIIS also common. Additional available techniques include ‘backwards NYSIIS’, metaphone and double metaphone. The lack of data cleaning functionality in some packages tended to be the result of a design decision to split this functionality into a separate software package rather than a value judgement about its usefulness.

### Advantages of data cleaning

In a record linkage context, the aim of data cleaning is to improve linkage quality [18,34]– that is, reduce the number of false positives (two records incorrectly identified as belonging to the same person) and false negatives (two records incorrectly identified as not belonging to the same person). Without data cleaning, many true matches would not be found, as the associated attributes would not be sufficiently similar [35].

Despite its widespread availability in linkage software packages, its use by numerous linkage groups, and its recognition as a key step in the record linkage process, the record linkage literature has not extensively explored data cleaning *in its own right*. Particular methods of cleaning data variables have been evaluated previously. Churches et al. [25] compared rule based methods of name and address standardisation to methods based on probabilistic models, finding more accurate address information when cleaned using probabilistic models. Wilson [36] compared phonetic algorithms and hand curated mappings on a genealogical database, finding the hand-curated mappings more appropriate for name matching. To our knowledge there has been no systematic investigation of the extent to which data cleaning improves linkage quality, or which techniques are most effective.

### Objectives

Implicit in the data cleaning process is the assumption that data cleaning will improve linkage quality. However there is limited literature that has quantified the extent of improvement arising from data cleaning. Moreover, little is known about the relative effectiveness of various techniques. The current study attempts to answer these questions through a systematic investigation of the effect of data cleaning on linkage quality using two datasets – a ‘synthetic’ dataset and a large-scale ‘real world’ administrative dataset.

Since real world datasets for which the ‘answers’ are known are both difficult to source and virtually impossible to share, we opted to generate and use a synthetic dataset. The synthetic data files contain artificially created records that have characteristics that closely resemble the attributes of real world datasets. Such datasets are typically use in benchmarking or systems testing.

## Methods

**Figure 1:**
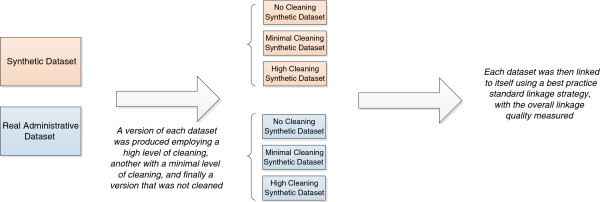
Road map for measuring overall linkage quality.

This study aimed to investigate both the overall combined effect of data cleaning, as well as the individual effects of specific data cleaning techniques. Firstly to investigate the overall quality, a highly cleaned, a minimally cleaned, and an uncleaned version of each of the two datasets was produced. These were each internally linked, with the resulting linkage quality measured. To investigate the effect of specific data cleaning techniques, the relative improvement of each transformation on the above datasets was measured and averaged Figure 1.

### Datasets

The synthetically generated data set consisted of 400,000 records, containing multiple records belonging to the same person. The synthetic data was generated using an amended version of the FEBRL data generator [37]. As a first step, the generator creates a user specified number of original records. These are created randomly, based on frequency lookup tables. Duplicate records are created in a second step, based on the original records. Duplicate records are created by randomly selecting an original record, then randomly choosing the number of duplicates to be created from it, and then randomly introducing errors according to user-specified parameters. An additional probability distribution specifies how likely data items or attributes are selected for introducing errors (it is possible for data items to have no errors at all).

The synthetic data file was based on frequency distributions obtained from the Western Australian electoral roll. As voting is compulsory in Australia, the electoral rolls are highly representative of the population. To avoid the potential of identifying individuals from the electoral data, the frequency list was truncated so that frequency counts below five were excluded.

Each record in the dataset comprised the following data items: surname, first name, sex, date of birth and postcode. Records in each dataset were generated with errors typically found in administrative data. Ascertaining representative rates of different types of errors such as duplications, omissions, phonetic alterations and lexical errors involved abstracting errors manually from a number of real world datasets and extrapolating these to the artificial data. Real world errors were applied to the synthetic data using user-specified parameters which are part of the Febrl data generator. Errors in the final dataset included the use of equivalent names, phonetic spellings, hyphenated names, first and last name reversals, change of surname, partial matches, typographical errors, incomplete or inaccurate addresses (postcode only) and change of address (postcode only). As Table 2 demonstrates, the synthetic datasets were highly representative of the source population.

**Table 2:** A comparison of the most common fields in the created synthetic data and the original data it was based on

**Surname (top 5)**	**Synthetic** **Per cent**	**Original** **Per cent**	**Male forename (top 5)**	**Synthetic** **Per cent**	**Original** **Per cent**
Missing value	1.98		Missing value	1.99	
Smith	0.92	0.94	John	3.44	3.47
Jones	0.55	0.55	David	3.09	3.09
Brown	0.46	0.46	Michael	2.95	2.95
Williams	0.46	0.46	Peter	2.87	2.88
Taylor	0.44	0.44	Robert	2.47	2.47
**Female forename (top 5)**	**Synthetic** **Per cent**	**Original** **Per cent**	**Postcode (top 5)**	**Synthetic** **Per cent**	**Original** **Per cent**
					
Missing value	1.99		Missing value	1.01	
Margaret	1.57	1.56	6210	2.84	2.84
Susan	1.35	1.34	6163	2.33	2.34
Patricia	1.22	1.22	6027	2.06	2.05
Jennifer	1.19	1.20	6155	2.02	2.02
Elizabeth	1.05	1.05	6065	2.00	1.98

This dataset had previously been used for an evaluation of linkage software [38]. An advantage to the use of synthetic datasets is that they are transportable, and so allow easier validation, and the ‘answers’ as to which records belong to the same person are available, unlike in real administrative data. This dataset is freely available (see Additional file 1).

Ten years of ‘real world’ hospital admissions data was sourced from one Australian state. This consisted of almost 7 million records. This dataset comprised the following fields: first name, middle name, surname, date of birth, sex, address, suburb, postcode and state. This data had previously been linked to a very high standard using probabilistic linkage along with a rigorous manual review of created links, and a quality assurance program to analyse and manually review likely errors. Based on quality assurance procedures, the estimated error rate of this linkage is 0.3% [39]. Furthermore, these links have been validated through this datasets use in a large number of research projects and published research articles [1]. The links created during this original linkage allowed us to evaluate our linkage quality in comparison.

Both synthetic data and real administrative data have advantages and disadvantages comparison data sets. Synthetic data may not manage to capture all the complexity of errors that real administrative data can. Using real administrative data requires relying on the results of previous linkages as a standard by which to compare which may not be entirely accurate, whereas synthetic data gives a known, accurate standard. By using both of these datasets in our analysis, we hope to avoid both of these issues, and gain the best of both worlds.

### Cleaning techniques

For each dataset, two sets of cleaned variables were computed – a minimally cleaned set and a heavily cleaned set. Information on the specific techniques used in each dataset can be found in Table 3. The generation of some variables required the creation of additional lookup tables: a nickname table, and a sex imputation table.

**Table 3:** Specific data cleaning techniques used on each dataset

**Synthetic data**		
**Fields available for linkage: forename, surname, date of birth, sex, postcode**		
**No cleaning**	**Minimal cleaning**	**High cleaning**
**Reformat values:**	**Reformat values:**	**Reformat values:**
Not required	Not required	Not required
	**Remove alt. missing values and uninformative values:**	**Remove alt. missing values and uninformative values:**
	Invalid dates of birth removed	Invalid dates of birth removed
	Invalid postal code values removed	Invalid post code values removed
	**Remove punctuation:**	**Remove punctuation:**
	Both forename and surname fields had all punctuation and spaces removed	Both forename and surname fields had all punctuation and spaces removed
		**Nickname lookup:**
		Nicknames were changed to their more common variant.
		**Sex Imputation**
		Records with missing sex had a value imputed based on their first name.
**Hospital admissions data**		
**Fields available for linkage: forename, middle name, surname, sex, date of birth, address, suburb, postcode, state**		
**No cleaning**	**Minimal cleaning**	**High cleaning**
**Reformat values:**	**Reformat values:**	**Reformat values:**
Date of birth reformatted.	Date of birth reformatted	Date of birth reformatted.
	**Remove alt. missing values and uninformative values:**	**Remove alt. missing values and uninformative values:**
	Invalid dates of birth were removed	Invalid dates of birth were removed
	Invalid postcode values were removed (‘9999’ etc.)	Invalid postcode values were removed (‘9999’ etc.)
	Uninformative address and suburb values removed (‘NO FIXED ADDRESS’, ‘UNKNOWN’ etc.)	Uninformative address and suburb values removed (‘NO FIXED ADDRESS’, ‘UNKNOWN’ etc.)
	Birth information encoded in first name removed (‘TWIN ONE OF MARTHA’ etc.)	Birth information encoded in first name removed (‘TWIN ONE OF MARTHA’ etc.)
	**Remove punctuation:**	**Remove punctuation:**
	Forename, middle name surname and suburb fields had all punctuation and spaces removed	Forename, middle name surname and suburb fields had all punctuation and spaces removed
		**Nickname lookup:**
		Nicknames were changed to their more common variant.

A nickname lookup table was developed based on similar nickname lookup tables found in linkage packages and as used by Australian linkage units. A sex imputation table was developed by examining the frequency of each given name in the data files and calculating the probability of the person being male or female. A record with a missing sex value was then given the most common gender value for this name.

### Linkage strategy

The linkage strategy chosen was based on a previously published default strategy used for an evaluation of linkage software [38]. A probabilistic linkage approach was used with two blocks (Soundex of surname with first initial, and date of birth) and all possible comparison variables were computed in each block. A String similarity measure (the Jaro-Winkler string comparator [40]) was used for all alphabetic variables (names, address and suburb) with exact matches being carried out on all other variables. Day, month and year of birth were all compared separately. Correct agreement and disagreement weights for probabilistic linkage [41] were calculated for each variable and used in linkage. The threshold setting was adjusted multiple times with the linkage quality computed for each adjustment, with the highest result (i.e. the largest F-measure) reported. The threshold was adjusted in both directions in increments of 0.5, until it was clear all future adjustments would continue to worsen the F-measure. This linkage strategy was based on a previously published ‘default’ linkage strategy [38].

### Linkage methods

As probabilistic record linkage techniques provide robust matching results for data which contain inconsistencies or incomplete data, these have been used throughout the study to match both the synthetic and ‘real world’ data sets. Following the traditional probabilistic linkage approach, pairs of records were compared and classified as matches if the matching score is above the threshold.

To calculate the matching score reached by a pair of records, each field (for instance first name or postcode) has been compared. Scores for each individual field were computed using agreement and disagreement weights. The agreement weight expresses the likelihood that records which belong to the same person have the same value for this field. The disagreement weight expresses the likelihood that records which do not belong to the same person have the same value on this field. The sum of these individual field scores has been computed and compared to the matching threshold to determine matches or non-matches [15].

### Linkage engine

BigMatch, developed by the US Bureau of Census [42] was used as the linkage engine for the analysis. BigMatch was chosen as it is fast, can handle large volumes, has a transparent linkage process based on probabilistic methods, and importantly, does not contain any automatic inbuilt data cleaning. The software had previously been evaluated and found to perform well against other linkage software packages [38].

### Measuring linkage quality

There are two types of errors that can be made in record linkage. Firstly there are incorrect matches, whereby two records are designated as belonging to the same person when they should not be (a false positive). Secondly there are missed matches, whereby two records are not designated as belonging to the same person when they should be (a false negative). These two types of errors can be measures as precision (the proportion of matches found that were correct) and recall (the proportion of correct matches that were found). A linkage with a high precision will have few false positives; similarly a linkage with high recall will have few false negatives. The F-measure of a linkage is the harmonic mean between precision and recall. This gives us a single equation with which we can compare linkage quality. These measures have been recommended as suitable for record linkage [43], and have been used previously in record linkage studies [38]. The calculations for these measures can be seen below.

$$\begin{array}{l} \text{Precision}=\frac{\text{Total}\;\text{number}\;\text{of}\;\text{correct}\;\text{pairs}\;\text{found}}{\text{Total}\;\text{number}\;\text{of}\;\text{pairs}\;\text{found}}\\ \text{Recall}=\frac{\text{Total}\;\text{number}\;\text{of}\;\mathrm{correct}\;\text{pairs}\;\mathrm{found}}{\text{Total}\;\text{number}\;\text{of}\;\mathrm{correct}\;\text{pairs}}\\ f-\text{measure}=\frac{2\times \text{Precision}\times \text{Recall}}{\text{Precision}+\text{Recall}} \end{array}$$

### Measuring the quality of a single variable

A similar approach to the one described above can be used when measuring the quality of a single variable. A variable which nearly always has the same value for all records belonging to the same person, but nearly always has a different value than all records belonging to other people, would be much more useful in the linkage process than one which seldom had these properties. Put in another way, a variable with a high precision (here measured as the proportion of times that two variables which have the same value belong to the same person) and a high recall (the proportion of times two records matching each other had the same value of the variable in question) will be more useful than one with lower precision and recall.

As some data cleaning techniques may increase precision and lower recall, we can determine which technique will have the overall best effect on predictive accuracy by using the F-measure of these two values. Furthermore we can measure the relative improvement of a data cleaning technique by comparing its individual F-measure before and after data cleaning.

## Results

The overall linkage quality results can be seen in Table 4. This represents the highest possible F-measure in each cleaning condition after testing multiple thresholds. The differences found when manipulating the level of data cleaning were very small. For both synthetic and hospital admissions data, a high level of data cleaning resulted in a decrease in linkage quality. Minimal cleaning resulted in a slight decrease in linkage quality for synthetic data, while remaining the same for hospital admissions data.

**Table 4:** Overall linkage quality results

**Synthetic data**	
	**F-measure**
No cleaning	0.883
Minimal cleaning	0.882
High cleaning	0.875
**Hospital admissions data**	
	**F-measure**
No cleaning	0.993
Minimal cleaning	0.993
High cleaning	0.992

Data cleaning techniques were further investigated to determine their individual effect in improving or decreasing linkage quality. Each variable had its predictive ability determined by calculating its own precision, recall and F-measure, where two values were said to match if they were exactly the same. The percentage difference in predictive ability between the cleaned variables and the original variables was then computed, with the average percentage change for each cleaning technique shown in Table 5. As there were no missing values for sex in the hospital admissions data, this technique was not used.

**Table 5:** Improvement in predictive ability of data cleaning techniques

	**Hospital admissions data**	**Synthetic data**
Remove punctuation	−^a^0.08%	+0.08%
Remove alt. missing values	+0.5%	0%
Nickname lookup	−28%	−33%
Sex Imputation	NA	−5%

^a^ Negative sign (-) refers to decrease in predictive ability, positive sign (+) refers to increase in predictive ability compared to baseline.

While removing missing values and uninformative values seemed to increased predictive ability, all other techniques displayed mixed or worse results. Using name variables that had nicknames and diminutive names replaced with their original names resulted in a large 30% decrease in that variable’s predictive value.

A sample of the precision and recall of the variables used is shown in Table 5. For individual transformations, the amount of correct matches found typically increases with data cleaning (increased recall), while the number of incorrect matches found also increases, resulting in lower precision. In general, the decrease in precision more than offsets the increase in recall, resulting in a decreased overall result. For instance, while the Soundex of surname (Table 6) resulted in an increase in the amount of correct matches found compared to the original surname field (from 98.8% to 99.4%, an increase of 0.6%), the percentage of matches found that were correct dropped 65% from 2.53% to 0.88%. This pattern is seen for most other transformations, and appears to be the reason for the decrease in linkage quality.

**Table 6:** Examples of single variable changes in predictive ability for individual cleaning techniques in hospital admission data

**Hospital admissions data**			
	**Precision**	**Recall**	**F-measure**
***Percentage difference from original variable***			
Given name original	0.006575	0.946085	0.013059
Given name with removed punctuation	0.006573*↓*^*b*^*0.03%*	0.947188*↑0.11%*	0.013056*↓0.02%*
Given name with nicknames removed	0.004357*↓33.7%*	0.953738*↑0.81%*	0.008675*↓33.5%*
Surname original	0.025265	0.98824	0.049271
Soundex of surname	0.008845*↓65%*	0.994926*↑0.67%*	0.017533*↓64.4%*
Address original	0.687066	0.669649	0.678246
Address with alternate missing values and uninformative values removed	0.687398*↑0.05%*	0.709426*↑5.9%*	0.698238*↑2.9%*

^b^ Down arrow symbol (*↓*) refers to decreased percentage change, up arrow (*↑*) refers to increased percentage change.

## Discussion

Overall, it was found that the effect of data cleaning on linkage quality was very small. If there was any effect at all, it appeared to decrease linkage quality. While some techniques led to small improvements, many others led to a large decrease in quality.

These results were not as expected. Data cleaning is assumed to improve data quality and thus to increase linkage quality. Examining the effect individual transformations had on a single variable’s predictive ability allows us to explain why this occurred. While the number of correct matches that were brought together increased with data cleaning, the number of incorrect matches also increased, in most cases dramatically. By removing the variability between records we are reducing our ability to distinguish one record from another.

Data cleaning techniques typically reduce the variability between values of the field in question. By removing nicknames, a smaller variety of names will be found in the dataset. By removing differences created by punctuation, this variability will be removed. As anticipated [7] this leads to a greater number of correct matches found; however this also leads to the identification of more incorrect matches.

### Strengths and limitations

Given the acceptance of data cleaning as an integral part of the linkage process, it was assumed that data cleaning would improve quality in general. The results obtained appear to contradict the conventional wisdom that data cleaning is a worthwhile procedure due to its ability to improve linkage quality.

Through the use of multiple representative datasets and the analysis of both linkage quality and individual transformations, these results seem robust. Measuring the effect of data cleaning in linkage is complex, as there are a multitude of parameters which can be altered that could affect the outcome of linkage quality. A potential concern is that some untested threshold value or other linkage parameter changes could drastically change these results. However, when analysed on their own, individual variables showed decreased predictive ability. If we accept that record linkage variables are independent (something which is an assumption of probabilistic record linkage) then it seems unlikely that any changes to linkage parameters will lead to linkage quality greater than that found in uncleaned data. On the other hand, the independence of variables used in linkage is often questionable, in which case the lower predictive ability of the individual variables is at the very least supportive of our conclusion.

The linkage strategy adopted here made heavy use of string similarity metrics. String similarity metrics may reduce the need for data cleaning, as they allow finer grained measures of similarity compared to exact matching, where variables with very slight differences will be treated as non-matches. A linkage strategy using exact matching only will have more need for data cleaning to bring correct records together, and this linkage strategy was not tested. However, the analysis of predictive ability of individual variables and their cleaned versions was carried out with exact matching only, which showed a decrease in predictive ability. This suggests data cleaning would not affect results any differently for those using an exact matching linkage strategy.

The linkages conducted simply replaced the original variables with the cleaned variables. An alternative method may be to use both the original and cleaned versions as variables in linkage. While this method violates the assumptions of independence underlying probabilistic record linkage [41], linkage variables are almost never independent, and such techniques have been implemented in some linkage packages. Further work would be required to determine the effect of using cleaned variables in conjunction with original uncleaned variables.

The f-measure was used as the sole measure of linkage quality. An underlying assumption of using this measure is that a single false positive is as equivalently undesirable as a single false negative. While this seems a sensible starting point, it should be noted that in numerous practical applications of record linkage this is not the case. For instance, if linking registry information to inform patients of their condition, it is much more important to reduce false negatives than false positives. Further analysis using additional metrics may be required to ensure these results hold using other linkage quality metrics. The key reason why cleaning failed to improve quality was the reduced variability of each field. Other data cleaning techniques not investigated here such as address standardisation increase the number of variables available for comparison and these techniques may improve quality.

### Avenues for further research

From this work it is clear that data cleaning does not always lead to increased linkage quality. Without further testing on a wide variety of datasets, it is hard to draw any further conclusions about the use of data cleaning in record linkage. Repeating this research on a wide variety of datasets is important. Further research into the use of cleaned as well as uncleaned variables together in the same linkage, into the use of further cleaning technique such as name and address standardisation is required. This research suggests that there are some situations where data cleaning transformations are helpful and others where they are not – determining a way of identifying when a transformation is likely to be helpful would be an important and useful finding.

## Conclusion

Data cleaning encompasses a variety of techniques which will be appropriate in specific circumstances. Care should be taken when using these techniques.

## Competing interests

All researchers involved in this study are employed by an Australian university. As with all Australian universities, the publication of work in a peer reviewed journal will result in credit being received.

## Authors’ contributions

Initial idea for research developed by AF. Linkage and analysis conducted by SR, with input from AF, JS and JB. SR drafted the manuscript with JS, JB and AF all providing substantial contributions. All authors read and approved the final manuscript.

## Pre-publication history

The pre-publication history for this paper can be accessed here:

http://www.biomedcentral.com/1472-6947/13/64/prepub

## Supplementary Material

Additional file 1 **Contains the synthetic data used in this paper.** This file is in comma separated, delimited format and is viewable in Microsoft Excel or any text editor. The features of this dataset are described more fully in the manuscript. Click here for file
